# Eutrophication: Early warning signals, ecosystem-level and societal responses, and ways forward: *This article belongs to* Ambio’*s 50th Anniversary Collection. Theme: Eutrophication*

**DOI:** 10.1007/s13280-020-01432-7

**Published:** 2021-02-03

**Authors:** Erik Bonsdorff

**Affiliations:** Environmental and Marine Biology, Åbo Akademi University, BioCity, 20500 Turku, Finland

**Keywords:** Ecosystem-based management, Eutrophication, Global change, Nitrogen, Nutrient loading, Phosphorus

## Abstract

Eutrophication, i.e. nutrient over-enrichment, has been a topic for academic and societal debate for the past five decades both on land and in aquatic systems fed by nutrients as diffuse loading from agricultural lands and as wastewater from industrial and municipal point-sources. The use of nutrients (primarily nitrogen and phosphorus) in excess became a problem with the onset of large-scale production and use of artificial fertilizers after World War II, and the effects on the aquatic environment became obvious some two to three decades later. In this Perspective, four seminal papers on eutrophication are discussed in light of the current knowledge of the problem, including future perspectives and outlooks in the light of global climate change and the demand for science-based holistic ecosystem-level policies and management options.

## Eutrophication as a regional and global driver of ecosystem change

Eutrophication, i.e. nutrient over-enrichment, has been a topic for academic and societal debate for the past five decades, both on land (agriculture and forestry) and in aquatic systems. It is fed by nutrients in the form of diffuse agricultural loading and from point-sources of industrial and municipal wastewaters. The use of nutrients (primarily nitrogen and phosphorus) in excess became a problem with the onset of large-scale production and use of artificial fertilizers after World War II, and the effects on the aquatic environment became obvious some two to three decades later (Cloern 1991; Reusch et al. 2018). Industrialized agriculture and forestry (including large-scale logging, clear-cutting, and mechanical reworking of the topsoil), as well as the draining of wetlands both inland and along the coasts, the global expansion of coastal aquaculture, and the ever increasing burning of fossil fuels, have increased the nutrient load on the aquatic environment (streams and rivers, lakes, and marine coastal and subsequently offshore waters). Rapid industrialization and urbanization have also contributed substantially to local, regional and gradually even world-wide spreading of harmful algal blooms, hypoxic events and large-scale fish-kills (Diaz and Rosenberg 2008; Rabalais et al. 2009; Kahru et al. 2020).

By the 1970s, the negative consequences of eutrophication had become obvious for environment and people, and measures had begun to be taken. There was an increased awareness of the simultaneously occurring negative consequences of other pollutants, habitat degradation, and of over-fishing in increasing areas of the global seas. Human awareness was primarily driven by issues related to human health and by the rapid decline in the populations of some charismatic species (mostly top predators, such as seals and other marine mammals, eagles and other large birds). Point-source pollution became the first target for improvement, and by the end of the 1970s to the mid 1980s, wastewater treatment plants were operating in many industrialized countries. Nutrient concentrations in effluents gradually decreased, as there was a shift in both technology and paradigm (from the classic ‘dilution is the solution to pollution’ to real reductions in the nutrient load on the aquatic environment). In the case of eutrophication, rapid progress was made in reducing phosphorus in urban and industrial waste waters. In contrast, nitrogen emissions remained high for one or two decades longer and still remain problematic, as the cost of further nitrogen reduction is sometimes considered higher than the benefit to society. The Baltic Sea is often referred to as one of the most polluted marine regions of the planet and can thus be taken as a prime lesson for other coastal and marginal sea regions (HELCOM 2013; Reusch et al. 2018). Here, the first significant ecological signs of recovery can be seen today, some 30 years after the start of reduction of nutrient inflow to the sea (Heiskanen et al. 2019). Thus, the gradual multi-decade transition from unrestricted stress by nutrient over-enrichment to changes in the functioning of ecosystems to partial recovery that included reduced inflow of nutrients, reduced nutrient concentrations in the environment, and significant change of the ecosystem-functioning could be demonstrated. Cloern (1991) illustrated the relationships between nutrient loading and long-term ecosystem responses both in the pelagic and the sedimentary systems on a global scale. Nixon (1995) provided a descriptive analysis of coastal eutrophication (defined as an increase in the rate of supply of organic matter to the aquatic environment, i.e. one step beyond the traditional definitions based on direct and indirect nutrient loads and concentrations in the environment). The large-scale overviews by Cloern (1991) and Nixon (1995) were developed further by Duarte et al. (2009), in an analysis showing that no direct (or at best a very slow) return to the pre-eutrophication pristine stage in a marine system can be expected. There may rather be multiple successive stable points following abatement of nutrient enrichment. Carstensen et al. (2011) further showed that most coastal ecosystems display a non-linear behavior to changing nutrient conditions, indicating the need for innovative and novel adaptation in the management of marine eutrophication. Today we know that the potential resilience of the ecosystem, and the recovery from the negative impact of eutrophication can be jeopardized by the escalating global climate change (Meier et al. 2012), which affects boreal and arctic coastal systems more severely than other aquatic ecosystems (Halpern et al. 2008; Reusch et al. 2018).

Since its start as a scientific journal in 1971, the journal *Ambio* has provided a forum for both academic and societal debate on central issues relating to the human–environment, including how society might tackle and solve these problems in sustainable ways. Among the topics raised in *Ambio* over the past decades, the negative consequences of eutrophication of the aquatic environment has been the subject of numerous papers (both based on specific case studies, and a variety of overview- and review papers, including a special issue on marine eutrophication in 1990), many of them highly cited. For the current thematic overview including perspectives and outlooks, four seminal and highly cited papers published in *Ambio* were selected by the Editorial Board: (1) Elmgren (1989) dealing with Man’s impact on the marine environment with an emphasis on the Baltic Sea, (2) Caraco and Cole (1999) with a focus on human nitrate export into riverine systems and their transport of nutrients to larger aquatic systems, (3) Galloway and Cowling (2002) providing a historic perspective on how increasing human use of reactive nitrogen has gradually become an environmental problem over the last two centuries, and (4) Matson et al. (2002) who focused on nutrient enrichment of terrestrial ecosystems, in contrast to the traditional way of viewing eutrophication as largely a problem for the aquatic environment.

## The Baltic Sea as a warning

The main emphasis in this overview is on the Baltic Sea, as this region is arguably the best studied marine ecosystem in the world when it comes to artificial human stress and the potential for recovery based on science-based policy-decisions and legalized management strategies (Reusch et al. 2018).

In the first of these influential papers, Elmgren (1989, with over 460 citations as of October 2020) made an important contribution by summarizing and analyzing human-induced stressors of the Baltic Sea during the twentieth century. He illustrated the rapid increase in nutrient concentration in most sub-basins of the sea, from the innermost low-saline brackish-water (even limnic) reaches in the North and East, to the almost fully marine regions in the South and West. This increase led to quantifiable and significant increases in primary production as increased productivity at most trophic levels throughout the marine food webs (measured as an increase in carbon flows, predating the definition for eutrophication presented by Nixon in 1995). The increase in nutrient loading also broadened the trophic pyramid. The widening of the food chain and the diversified basis for the increased productivity influence the ecosystem at large. This was partly counterbalanced by a thinning of the highest trophic levels through human overexploitation of the increased production of dominant fish species, and through the negative consequences on the aquatic ecosystem by toxic substances accumulating at the highest trophic levels (marine mammals, predatory fish and birds). Elmgren (1989) estimated that during most of the twentieth century (1900–1980), pelagic primary production had increased approximately 30–70%, depending on region along the steep Baltic Sea environmental gradients (Reusch et al. 2018). Simultaneously, sedimentation of organic matter to the sea floor was estimated to have increased dramatically by 70–190%, contributing to the spreading of hypoxia and anoxia in both coastal and open waters of the Baltic Sea, as a consequence of increased oxygen demand in the near-bottom water layers of this strongly stratified sea (Conley et al. 2002; Carstensen et al. 2014). Elmgren (1989) also found that zoobenthic biomass had doubled above the halocline (i.e. closer to the coasts fueled by the increased primary production and sedimentation rates), whereas open-water pelagic zooplankton biomass was estimated to have increased by up to 25%. During the same period fish catches in the Baltic Sea had increased by an order of magnitude, partly explained by the broader base of the food web and increased overall productivity, and partly by the increased efficiency of the commercial fisheries. In an era when humans considered other top consumers as competitors for fish in the marine realm (Hansson et al. 2018), Elmgren (1989) could show that in fact the proportion of the basic resources appropriated for human use had increased, but decreased for marine mammals, which by the 1980s had reached their lowest abundances due to hunting and to pollutants affecting reproductive success. Since then a gradual recovery of some marine top consumers (e.g. seals, eagles, cormorants) has been recorded (Hansson et al. 2018; Reusch et al. 2018), and the basic food web structure has remained relatively stable in spite of major regime shifts among top consumers in the ecosystem (Yletyinen et al. 2016). In a follow-up paper, Elmgren (2001) further underlined the concern of several earlier studies for the resilience of the Baltic Sea ecosystem, illustrating that an ecosystem may bounce back to some extent, provided conditions are consistently improved through long-term investments and commitments, but emphasizing that society should view environmental management decisions as experiments, to be monitored, learned from, and then continuously modified as needed based on science-based environmental advice (for recent analyses, see Andersen et al. 2011, 2017). This approach has successfully been adopted for specific regional studies (see HELCOM 2013 for the open Baltic Sea, and Riemann et al. 2016 for the Danish coastal waters), and conceptually developed as general management tools for marine coastal waters in a broad perspective (Elliott et al. 2017), illustrating positive development for the Baltic Sea ecosystem (Reusch et al. 2018; Heiskanen et al. 2019).

## Nitrogen as a driver of global environmental change

While Elmgren (1989) focused on an entire marine ecosystem under human stress, with eutrophication as an overriding stress factor, Caraco and Cole (1999, with over 410 citations by October 2020) modeled the export of nitrate to the global coastal ocean by studying 35 major river systems from all inhabited continents on Earth. They found large variability in the export of N, indicating that the main factor determining N export was human activity in the watershed of the river basins studied. Over 50% of the variability was explained by human population density alone. An intriguing finding was that the share of man-induced nitrogen (both fertilizers and point-source pollution) also increased with increasing human population, i.e. with increasing population density in the watershed the share of human-derived nutrient input increased almost exponentially. Caraco and Cole (1999) thus managed to explain over 80% of the more than 1000-fold variability in nitrate export from the different regions of the world. They also indicated that the riverine export may further increase in the future due to increased coastal populations and/or decreased watershed retention. Both of these suggestions seem valid today, as global warming affects precipitation and runoff, and human populations increase along the major riverine systems and coastal regions of the world (Paasche and Bonsdorff 2018). Ecosystem-level impacts are expected in coastal waters around the world, unless multi-stressor impacts are managed simultaneously and consistently.

Where Caraco and Cole (1999) used a model to estimate current (late 1990s) nitrogen exports from the major riverine systems on different continents and under varying population and climate regimes, Galloway and Cowling (2002, cited over 1660 times by October 2020) analyzed the use and misuse of nitrogen over the 200 year period since the element nitrogen was discovered in the late eighteenth century. They showed the extremely rapid exponential increase in the annual production of reactive nitrogen since the 1950s, as industrial processes were developed and refined. One focus in the analysis by Galloway and Cowling (2002) is the major importance of the Haber–Bosch process for manufacturing artificial nitrogen fertilizer. They convincingly illustrated the drastic shift in the global nitrogen budget by comparing 1890 and 1990, and they also showed the significant difference in efficiency of using the fertilizer for a vegetarian or a carnivorous diet. They foreshadowed the global discussion going on today, including the current discussion of inefficient macro-grazers (cattle) contributing both to an over-use of nitrogen, and an increase in greenhouse gas emissions. From the global eutrophication perspective, a major knowledge gap identified by Galloway and Cowling (2002) concerned N-storage in the natural environmental reservoirs that could potentially lead to cascading effects on the ecosystem (which, incidentally, is the effect Elmgren 1989 demonstrated for the Baltic Sea as an overall consequence of regional nutrient over-enrichment). The foresight in the paper by Galloway and Cowling is impressive, with calls for action still not taken seriously in many parts of the world, with increasing eutrophication problems as a consequence. The application of lessons learned in one region for the benefit of other regions is one of the great challenges for the future management of the global environment (Reusch et al. 2018).

Galloway and Cowling (2002) analyzed the historic development of nitrogen impact on aquatic and terrestrial systems, driven largely by the industrial manufacture of artificial nitrogen fertilizer. Simultaneously, Matson et al. (2002, with over 715 citations as of October 2020) provided a comprehensive perspective into the nitrogen deposition on terrestrial ecosystems. Most people associate eutrophication with aquatic ecosystems, be it freshwater or marine, but in their interesting paper, Matson et al. (2002) discuss how much of the nitrogen used is actually incorporated in vegetation and soils, thus providing understanding of how a legacy of excess nitrogen is gradually being stored in the environment. They also show that although there is an initial retention of N in the soils, leaching into the watershed and subsequently into the aquatic ecosystems increases over time, causing what is today often called internal nutrient loading. This may feed internal loops of production in the marine ecosystem, as exemplified for the Baltic Sea by Vahtera et al. (2007), with couplings to other ecological processes that influence management options. Leaching of nitrogen from land is driven by the type of soil and by precipitation and hydrologic features of the terrestrial system bordering aquatic ones. These processes have been illustrated and verified for the Baltic Sea by McCrackin et al. (2018), who showed that the terrestrial soil-bound legacy of nutrients is huge and may slow ecosystem recovery for decades to come. Matson et al. (2002) further illustrate the relationships with soil acidification and aluminum toxicity, and how gaseous N is lost to the atmosphere. With a continuously increased demand for and use of artificial fertilizers, the entire global N-cycle has been altered (see also Galloway and Cowling 2002), which in turn has major impacts on biological diversity both on land and in the sea.

## Perspectives and outlook

The four seminal papers discussed above all deal with nutrients and eutrophication and stress two central issues important as management strategies are developed to minimize the negative consequences of eutrophication. The first is that further detailed and adaptive multidisciplinary research is needed to fully understand the general patterns, processes and impacts of both aquatic and terrestrial eutrophication, and second, that large-scale ecosystem-level effects of the multidecadal over-use of nutrients (both phosphorus and nitrogen) have had dire effects on the biota at all trophic levels of all studied ecosystems. In the light of current knowledge of the effects of global climate change, serious efforts must be made on different societal and environmental levels, and in various political and legislative arenas to counter the negative effects and impacts, and to avoid mega-scale ecosystem regime shifts and tipping-points with effects cascading through the trophic webs (Paerl and Paul 2012).

In order to fully comprehend the effects of eutrophication on large-scale systems, elaborate modeling approaches are needed, and in order for such efforts to be effective, long time-series of data are required and should continue to be collected in basic research and monitoring at all levels and in multiple ecosystems around the world (Reusch et al. 2018; Stenseth et al. 2020). A good example of how such data can be utilized is given by Gustafsson et al. (2012), who reconstructed the eutrophication process of the Baltic Sea for the period 1850–2006. This in turn has been used as a basis for an analysis of how best to combat eutrophication today (Meier et al. 2012). Utilizing current modeling tools in combination with adequate management options, realistic outcome-scenarios can be presented (Elliott et al. 2017; Heiskanen et al. 2019), and thus the concerns and pleas of Elmgren (1989, 2001) may be heard (Murray et al. 2019). In their current analysis, Murray et al. (2019) present alternative models and outcomes in relation to the updated HELCOM Baltic Sea Action Plan (HELCOM 2013). They clearly illustrate potential outcomes of various management schemes for the different sub-basins and regions of the Baltic Sea. Their main finding is that strong measures are needed regarding both nitrogen and phosphorus, and that recovery will take decades, but also that positive results regarding the eutrophication status of the Baltic Sea can be achieved, although the uncertainty of a changing climate looms over the best of predictions. Hence, there is both need and room for continued research into the issues of eutrophication, raised in *Ambio* over the past decades, as a basis for both scientific and societal debate and action.

With an increasing coupling to climate-driven global change, the entire oceanic system may ultimately provide severe and serious feedbacks to human society (Paasche and Bonsdorff 2018). In order to tackle or to minimize the impacts of such anthropogenically driven processes, lessons learned from studies of specific problems (eutrophication in this case) should be taken seriously, as human livelihood ultimately depends on the marine realm (Reusch et al. 2018; Stenseth et al. 2020). Until now (2020), the share of published contributions in the journal *Ambio* that combines ‘eutrophication’ and ‘climate/global change’ has been low (3.9% for the entire publication-period of 50 years of existence), although the number of papers on these topics individually has increased dramatically from 60 papers on eutrophication and 101 on climate/global change during the first 25 years (through 1995), to 169 and 510, respectively, in the 25 years from 1996 until October 2020. Only 33 papers in total combine eutrophication with climate/global change-related aspects (based on an analysis of titles, key words and abstracts using JSTOR and ISI Web of Science as sources), illustrating the need for research within this field. Hence, there is an increased need for dialogue and active collaboration between Science and Policy/Society, and eutrophication in the light of global change has become one example of this emerging multidisciplinary field, and future perspectives depend on our capability to integrate and amalgamate knowledge from cross-the-board perspectives and genuine collaborative efforts from specific to general aspects for the future (Rudolph et al. 2020). Schindler (2006) provided a good example of ways forward when understanding and tackling freshwater eutrophication, and Andersen et al. (2011, 2017) presented basin-wide comprehensive analyses and assessment schemes for eutrophication in the Baltic Sea, which were followed by an increased understanding of the potential recovery from eutrophication (Riemann et al. 2016; Heiskanen et al. 2019), and how the lessons learned over the twentieth century (Elmgren 1989, 2001) may in fact be developed into regional and global management options (Elliott et al. 2017), with implications for global networks of understanding and managing of the coastal oceans (Cloern et al. 2016; Reusch et al. 2018; Stenseth et al. 2020). Thus, it is evident that eutrophication can no longer be seen as an isolated source of environmental stress, but rather must be integrated in the scientific and societal debate of global climate change, posing massive challenges for the twenty-second century (Kahru et al. 2020; Rudolph et al. 2020).
